# Pyridoxal phosphatase is a novel cancer autoantigen in the central nervous system

**DOI:** 10.1038/sj.bjc.6602142

**Published:** 2004-09-28

**Authors:** A S Bøe, G Bredholt, P M Knappskog, A Storstein, C A Vedeler, E S Husebye

**Affiliations:** 1 Division of Endocrinology, Institute of Medicine, Haukeland University Hospital, N-5021 Bergen, Norway; 2 Center for Medical Genetics and Molecular Medicine, Haukeland University Hospital, Norway; 3 Department of Neurology, Haukeland University Hospital, N-5021 Bergen, Norway

**Keywords:** autoimmunity, pyridoxal 5′-phosphate, pyridoxal phosphatase

## Abstract

Autoantibodies against many proteins are common in sera from patients with various types of cancer. These antibodies are sometimes involved in the development of conditions associated with cancer, such as paraneoplastic neurologic disorders. We used a human brain cDNA expression library and serum from a paraneoplastic neurologic disorder patient to search for new autoantigens in the nervous system. Pyridoxal phosphatase was identified as a novel autoantigen. Expression studies showed that pyridoxal phosphatase was strongly expressed in various parts of the central nervous system. Sera contained antibodies against pyridoxal phosphatase in 22 of 243 (9.1%) patients with lung cancer and eight of 113 (7.1%) with other forms of cancer *vs* two of 88 (2.3%) healthy control subjects. In addition, 2–4% of patients with different autoimmune diseases had autoantibodies against pyridoxal phosphatase. None of the antipyridoxal phosphatase-positive patients were known to have a paraneoplastic neurologic disorder. Hence, autoantibodies against pyridoxal phosphatase correlate with cancer but not necessarily with the subset of patients with paraneoplastic neurological disorders although serum from such a patient was used to screen the cDNA library. This study showed that yet another enzyme involved in pyridoxal 5′-phosphate metabolism is an autoantigen. Thus, pyridoxal 5′-phosphate seems to be a common denominator for autoantigens involved in autoimmune diseases.

Antibodies against nontissue specific intracellular or cell-surface proteins are seen in several types of cancer, such as antibodies against p53 (Crawford *et al*, 1982), oncogene products such as ras (Cheever *et al*, 1995), cellular proteins such as p62 (Zhang *et al*, 1999) and cell cycle proteins such as cyclin B1 (Covini *et al*, 1997). Other malignant conditions are characterised by autoantibodies against tissue-restricted proteins, as seen in patients with paraneoplastic neurologic disorders (PND). These patients produce antibodies and cytotoxic T cells that target neuron-specific proteins abnormally expressed in non-neuronal tumours causing neuronal injury (Dalmau and Posner, 1997; Bataller and Dalmau, 2003). The pathogenic role of onconeuronal autoantibodies is often unclear. In other cases, such as Lambert–Eaton myasthenic syndrome, antibodies are directly involved in pathogenesis, such as by blocking voltage-gated calcium channels and causing neuromuscular conduction blockade (Lang *et al*, 1981).

Detection of circulating autoantibodies signals the presence of malignant disease, often at an early stage (Trivers *et al*, 1995, 1996 and assay of cancer-associated antibodies can be used in diagnosing cancer. Further, an immune response against the tumour should theoretically help in controlling tumour growth. Souza *et al* (2003) have recently shown that patients with previous autoimmune thyroid disease and well-differentiated thyroid cancer have a better prognosis than those without signs of thyroid autoimmunity.

By using serum from a patient with known PND, we screened a human brain cDNA expression library to identify novel cancer-related or paraneoplastic autoantigens. One of the positive clones was identified as pyridoxal phosphatase (PDXP) (GenBank accession number AY125047 and BC000320) (Jang *et al*, 2003). This enzyme dephosphorylates pyridoxal 5′-phosphate (PLP), an important cofactor for enzymes involved in vitamin B metabolism and in neurotransmitter biosynthesis.

## METHODS AND SUBJECTS

### Subjects

Sera were obtained from 243 patients with lung cancer, 113 with different other types of cancers, 47 with multiple sclerosis (MS), 47 with type 1 diabetes, 72 with autoimmune Addison's disease or autoimmune polyendocrine syndromes (APS) and 88 healthy blood donors.

### Screening of the cDNA expression library

A human brain *λ* TriplEx cDNA expression library (human brain 5′-Stretch Plus cDNA Library, Clontech, Palo Alto, CA, USA) was screened with serum from a patient with ovarian cancer and paraneoplastic cerebellar degeneration containing Yo antibodies using the method described by Rorsman *et al* (1995). Briefly, bacteria and the cDNA library were mixed on a Petri dish containing NZY agar. After about 3.5 h of culture, plaque appeared on the dish. Plaques were copied onto a Hybond C nitrocellulose membrane (Amersham Pharmacia Biotech, Uppsala, Sweden) containing isopropyl-*β*-D-thiogalactoside (Sigma-Aldrich, Steinheim, Germany). Sera and secondary alkaline phosphatase-conjugated antibodies were added to the filter, followed by 5-bromo-4-chloro-3-indolyl phosphate/nitro blue tetrazolium (BioRad, Sundbyberg, Sweden) to detect immune complexes. Positive clones were rescreened until pure isolates were obtained and thereafter sequenced and identified by a BLAST search (http://www.ncbi.nlm.nih.gov/BL AST). Full-length clones were ordered when needed from the I.M.A.G.E. Consortium (http://image.llnl.gov).

### Radioimmunoassay (RIA)

The full-length coding cDNA was amplified by polymerase chain reaction using a forward linker-primer containing an *Nde*I restriction site (5′-GGAATTCCATATGGCGCGCTGCGAGAGGCT-3′) and a reverse linker-primer containing a *Bam*HI restriction site (5′-GGCGGATCCGGCTCAGTCCTCCAACCCCTCTGTCAA-3′). The cDNA was then ligated into the *Nde*I and *Bam*HI restriction sites of pET-19b (Novagen Inc., Madison, USA) and expressed as a 10 × His-tag fusion protein in the *in vitro* TnT transcription–translation coupled (ITT) system from Promega (Promega, Leiden, the Netherlands) in the presence of [^35^S]methionine (cell labelling grade, Amersham Pharmacia Biotech) and screened by RIA (Falorni *et al*, 1995). Titre of antibodies was calculated as relative values compared with standard positive and negative controls ((cpm subject X–cpm negative standard)/(cpm positive standard–cpm negative standard) × 1000). The ‘normal level’ of autoantibodies against this protein in sera was estimated by calculating the mean of index values for 88 healthy blood donors and adding 3 standard deviations.

### Protein expression and purification

The protein was expressed as a 10 × His-tag fusion protein by the vector pET19b in the *Escherichia coli* strain Rosetta (DE3) pLysS (Novagen, Madison, WI, USA). In total, 500 ml of Luria-Bertani medium containing 50 *μ*g ml^−1^ ampicillin was inoculated with 4 ml of an overnight culture of bacteria generated from a single colony of freshly transformed cells and incubated at 37°C. The bacterial culture was grown at 37°C, and protein expression was induced with 1 mM isopropyl-*β*-D-thiogalactoside at an optical density at 600 nm of 0.9. The cells were harvested 3.5 h later by centrifugation. Inclusion bodies were purified in the presence of complete, EDTA-free Protease Inhibitor Cocktail Tablets (Roche, Basel, Switzerland) using the B-PER Bacterial Protein Extraction Reagent (Pierce, Rockford, IL, USA) according to the manufacturer's protocol. Inclusion bodies from a 75-ml culture were dissolved in 2 ml of 6 M guanidine-HCl, 20 mM NaH_2_PO_4_, 500 mM NaCl, pH 7.8 before adding 10 ml of 8 M urea, 20 mM NaH_2_PO_4_, 500 mM NaCl, pH 7.8 (buffer A). The solution was then centrifuged at 20 000 × **g** and filtered through a 0.45-*μ*m syringe filter followed by nickel chelate chromatography using the ÄKTAprime chromatography system and a 1-ml HiTrap Chelating HP column (Amersham Pharmacia Biotech) under denaturing conditions. Briefly, the solubilised inclusion bodies were loaded onto the column followed by washing with 40 column volumes of buffer A containing 20 mM imidazol. The protein was eluted at low pH by exchanging the wash buffer with a buffer containing 8 M urea, 20 mM NaH_2_PO_4_, 500 mM NaCl, pH 3.5 with a gradient from 0 to 100% over 20 column volumes. The protein purity was evaluated by sodium dodecyl sulfate–polyacrylamide gel elecrophoresis (SDS–PAGE) followed by Coomassie blue staining. The concentration was determined by the Bradford protein assay (BioRad) using bovine serum albumin (BSA) as standard according to the protocol of the manufacturer.

### Rabbit PDXP antiserum

Purified PDXP was dialysed against sterile phosphate-buffered saline (PBS). Two female outbred rabbits were immunised intradermally with about 50 *μ*g of purified protein emulsified 1 : 1 in Freund's complete adjuvant. The rabbits were boosted with about 50 *μ*g of protein emulsified 1 : 1 in Freund's incomplete adjuvant on days 14, 28, 56 and 86 after the primary immunisation. After reactivity towards the protein antigen was verified by RIA of radiolabelled protein expressed by ITT (described above) and by Western blot (described below), the rabbits were terminally bled on day 96 and serum was collected.

## WESTERN BLOT

Recombinant PDXP (about 15 *μ*g) was incubated in 4 × sample buffer and 10 × reducing agent and applied to the large well on a 4–12% NuPAGE MOPS Bis-Tris gel (Invitrogen, Merelbeke, Belgium). The molecule markers used were the high-range rainbow molecular weight marker (Amersham Pharmacia Biotech) for radiolabelled samples and the Seeblue Plus 2 prestained standard (Invitrogen) for nonlabelled samples. The proteins were blotted onto a Hybond P PVDF membrane (Amersham Pharmacia Biotech) and incubated with sera from 24 cancer patients that were positive for anti-PDXP in RIA or immunised rabbit-serum diluted 1 : 100 in blocking buffer (PBS with 4% fat-free dry milk) and then with alkaline phosphatase-conjugated rabbit anti-human IgG (Sigma-Aldrich) and goat anti-rabbit IgG (Southern Biotechnology, Birmingham, AL, USA), diluted 1 : 3000 in blocking buffer. The substrate for alkaline phosphatase was 5-bromo-4-chloro-3-indolyl phosphate/nitro blue tetrazolium (BioRad).

### Immunohistochemistry

We used autopsy material from a 69-year-old man, otherwise healthy, who had died from sudden cardiac arrest 24 h previously. Tissue from cerebrum, cerebellum, liver, kidney, spleen, heart, lung and muscle was taken from 2–3 locations in each organ, snap-frozen in isopenthane cooled in liquid nitrogen and stored at −80°C until use. A total of 8 *μ*m thick cryostat sections were incubated with the preimmunised or immunised rabbit sera against PDXP diluted 1 : 100 in PBS and then with Alexa Fluor goat anti-rabbit IgG (Molecular Probes, Eugene, OR, USA) diluted 1 : 100 in PBS. Analysis used a LEICA fluorescence microscope. In some experiments, sections of human brain were stained with rabbit anti-PDXP diluted 1 : 100 in PBS followed by peroxidase-labelled anti-rabbit antibody and analysis by light microscopy. Tissue sections were incubated without prior fixation, as we experienced that fixation with acetone or formaldehyde prior to primary incubation did not result in enhanced staining.

### Northern blot

In total, 20 *μ*g of total RNA from rat cerebellum purified by the RNeasy mini kit (Qiagen, Hilden, Germany), rat brain (Clontech) and of a pool of total RNA from a wide selection of human tissues (Human Reference total RNA; Clontech) were denatured, electrophoresed on a denaturing 1.2% formaldehyde agarose gel and blotted onto a Hybond N nylon membrane (Amersham Pharmacia Biotech) by capillary blotting in the presence of saline sodium citrate buffer (10 × SSC; 1.5 M NaCl, 0.15 M sodium citrate, pH 7.0). The membrane was washed in a buffer containing 1.6 × SSC and 0.2 M Tris-HCl pH 7.2 and baked at 80°C for 15 min, and the RNA was crosslinked to the membrane at 120 mJ using Stratalinker 1800 UV (Stratagene, La Jolla, CA, USA). Full-length PDXP cDNA (20 ng) was labelled with 4 *μ*l of *α*-^32^P-dCTP (3000 *μ*Ci mmol^−1^, Perkin-Elmer, Boston, MA, USA) using the Megaprime kit (Amersham Pharmacia Biotech) and purified over a Sephadex G-50 (Amersham Pharmacia Biotech). The blot was wetted in 2 × SSC and prehybridised for 1 h at 65°C in 16 ml of hybridisation buffer containing 2 mg ml^−1^ bovine serum albumin, 2 mg ml^−1^ Ficoll 400, 2 mg ml^−1^ polyvinylpyrrolidone K25, 0.5 M NaH_2_PO_4_, 14 mg ml^−1^ SDS, 1 mM EDTA, 5 mg of heat-denatured, sheared salmon sperm DNA (Sigma-Aldrich) and 30 *μ*g of heat-denatured human Cot-1 DNA (Invitrogen). The probe was denatured by heat in 1 ml of hybridisation buffer, added to the hybridisation solution used for prehybridisation and incubated with the membrane at 65°C overnight. The hybridised membrane was then washed four times at 65°C for 30 min in a washing buffer (20 mM NaH_2_PO_4_ pH 7.2 and 5 mg ml^−1^ SDS) and exposed to Kodak BioMax MS Film (Eastman Kodak Company, New Haven, CT, USA) at −80°C for 2 days using BioMax TranScreen HE intensifying screen (Eastman Kodak Company).

### Expression studies

Gene expression in human tissues of the isolated cDNA was determined by hybridising cDNA with the Multiple Tissue Expression Array (Clontech). Full-length coding cDNA was labelled with *α*-^32^P-dCTP, hybridised against the Multiple Tissue Expression Array and developed as described for Northern blots. In addition, rabbit PLP phosphatase antiserum (described above) diluted 1 : 500 was applied to the Newborn Rat Brain Protein Explorer (Alpha Diagnostic International, San Antonio, TX, USA), a commercial ready-made membrane containing brain proteins from rats. Hybridisations were detected using chemiluminescence following the instructions of the manufacturer, except that donkey anti-rabbit horseradish peroxidase-coupled IgG (H+L) (Jackson ImmunoResearch, West Grove, PA, USA) and the SuperSignal West Femto Maximum Sensitivity Substrate chemiluminescence kit (Pierce) were used for detection instead of the reagents in the kit. The membrane was exposed for 15 min on a IMAGE Station 2000R (Eastman Kodak Company).

### Statistical analysis

Differences between groups were assessed using the Pearson *χ*^2^ test.

### Ethical considerations

The study has been carried out with ethical committee approval. For animal experiments, United Kingdom co-ordinating committee on cancer research (UKCCCR) guidelines for the Welfare of Animals in Experimental Neoplasia were followed (Workman *et al*). The study was performed according to the Declaration of Helsinki.

## RESULTS

### Identification of a novel autoantigen

Screening the cDNA library with the PND serum revealed a positive clone that was isolated and sequenced. The nucleotide sequence showed that the clone was identical to a truncated form of PDXP that covered amino-acid codons 122–297 (GenBank accession number AY125047) (Fonda, 1992; Gao and Fonda, 1994a, 1994b, 1994c; Fonda and Zhang, 1995; Jang *et al*, 2003). Full-length cDNA for PDXP was ordered from the I.M.A.G.E Consortium and subcloned into the pET19b vector. The full-length coding sequence contained 891 nucleotides and encoded a protein of 297 amino acids with a molecular weight of 32 kDa.

### Expression of recombinant PDXP and production of antibodies against PDXP

**Figure 1:**
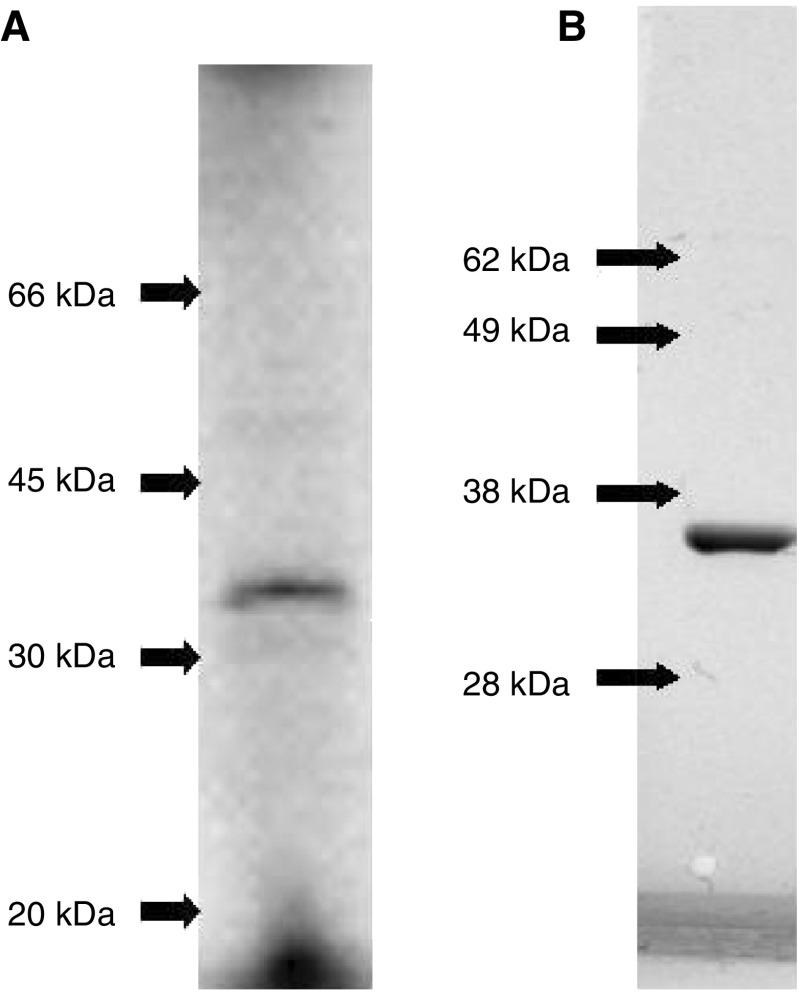
(**A**) SDS–PAGE of *in vitro* coupled transcribed and translated ^35^S-labelled PDXP. (**B**) SDS–PAGE of 2 *μ*g *E. coli*-expressed purified PDXP detected by Comassie blue staining.

We expressed PDXP as ^35^S-labelled protein using ITT (Figure 1A

). The protein was also expressed in *E. coli* and purified to about 95% as judged by SDS–PAGE (Figure 1B). The molecular weight of the ITT-expressed protein correlated with the weight of the bacterially expressed PDXP. Purified *E. coli*-expressed protein was used to immunise a rabbit in order to achieve antibodies against PDXP. One immunised rabbit produced a high-titre antiserum against PDXP as assessed by RIA and Western blot, whereas serum from the preimmunised rabbit did not bind the protein.

### Tissue expression of PDXP

**Figure 2:**
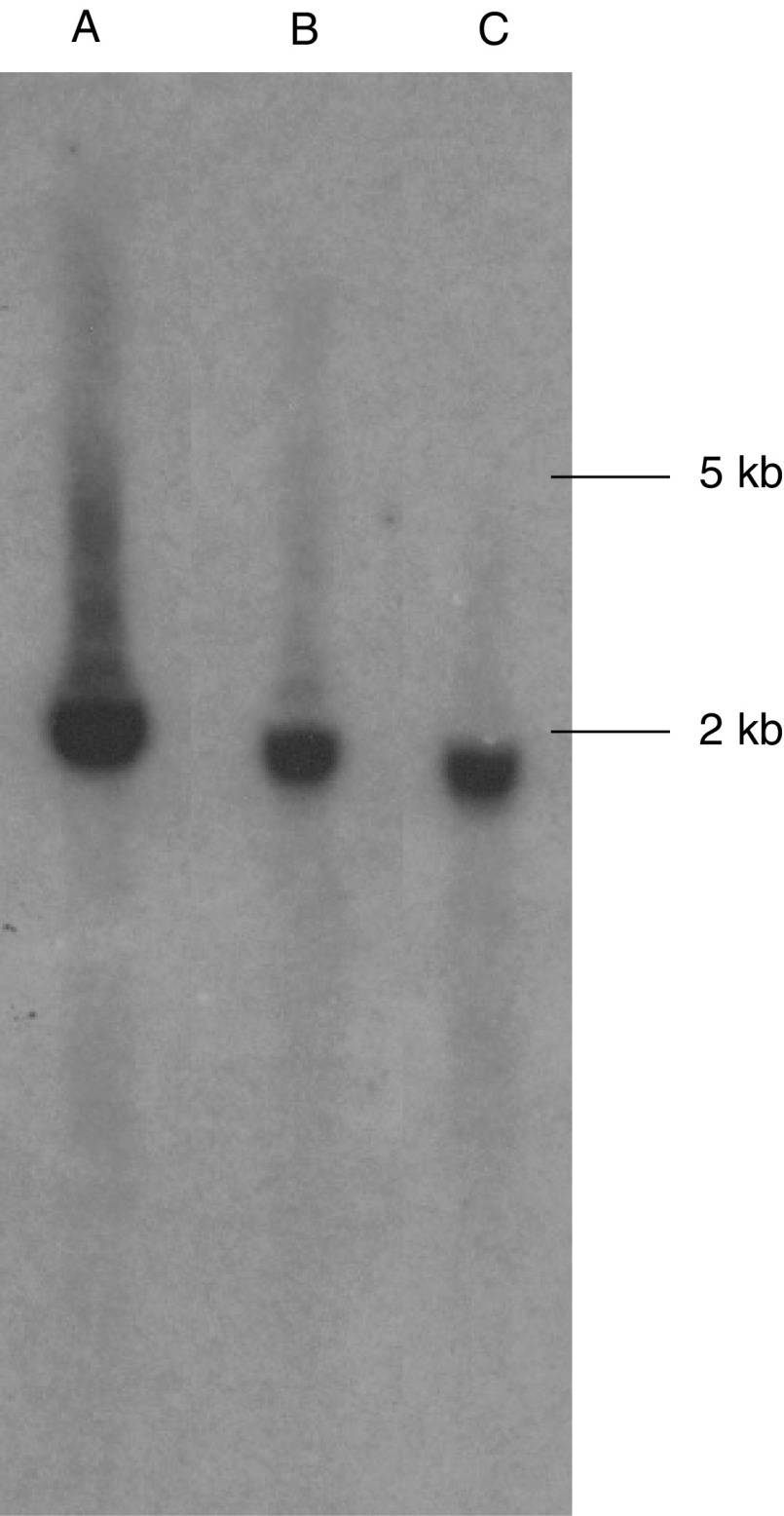
Northern blot of PDXP cDNA. The cDNA was labelled with *α*-^32^P-dCTP and hybridised against total RNA electrophoresed on a formaldehyde agarose gel. (A) Human reference total RNA. (B) Rat brain total RNA. (C) Rat cerebellar total RNA.

**Figure 3:**
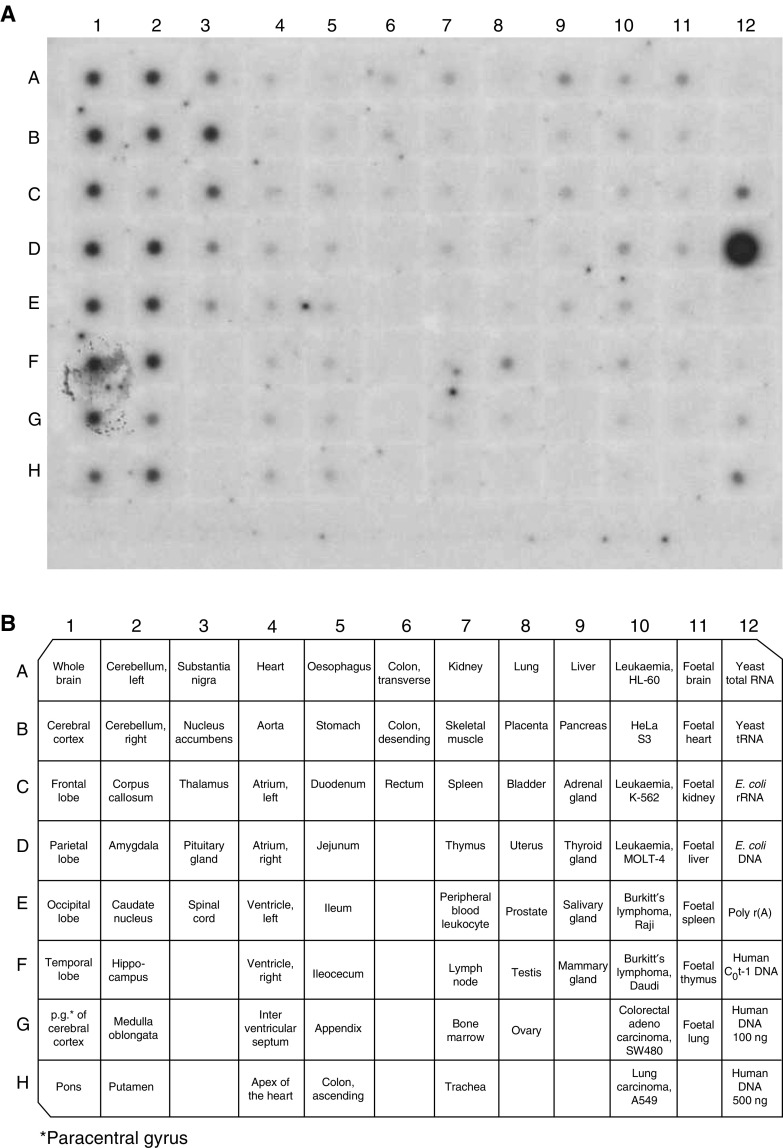
Expression of PDXP in various human normal tissues and cancer cell lines. The PDXP cDNA was labelled with *α*-^32^P-dCTP and hybridised against the Multiple Tissue Expression Array consisting of normalised, polyA RNA spotted on a nylon membrane as denoted in (**B**) PDXP transcripts are most abundant in the nervous system (columns 1–3) (**A**). The cDNA probe hybridised with *E. coli* DNA, probably due to several identical nucleotide stretches.

Northern blot of radioactive-labelled full-length PDXP against RNA pools of different origins revealed that the molecular weight of the messenger RNA was about 2 kb (Figure 2

) (Jang *et al*, 2003). The results from mRNA multitissue expression level analysis of PDXP using polyA RNA dot blot indicated that the PDXP gene was strongly expressed in different parts of the central nervous system and also to some extent in testis, liver, kidney and cancer cell lines (Figure 3

). Most of the other tissues represented on the blot also expressed PDXP but to a lesser degree than in the neuronal tissue. These results were supported by results obtained from analysis of the brain ReadyBlot explorer kit (data not shown).

### Autoantibodies against PDXP in patients with cancer, autoimmune disease and controls

**Figure 4:**
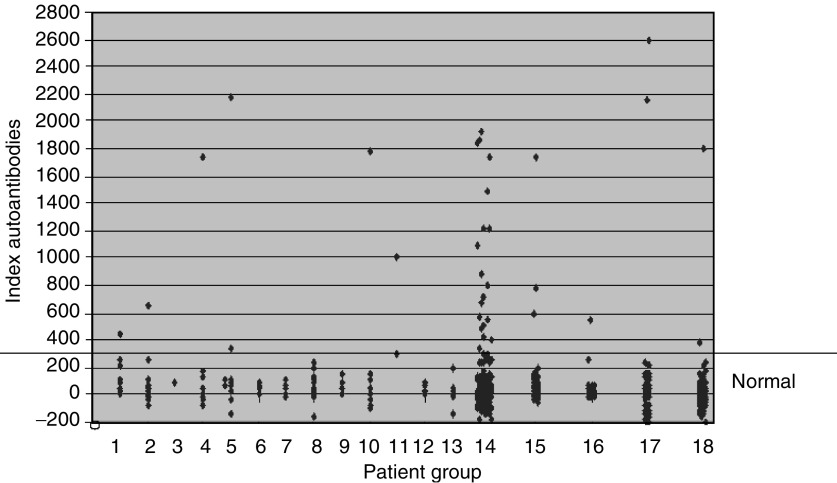
PDXP autoantibody analysis using immunoprecipitation assays. The figure shows the distribution of index values for the different groups of subjects used in this study. PDXP antibody reactivity is given as indices relative to a positive (patient serum) and negative control (pool of sera from healthy individuals) ((cpm subject X−cpm negative standard)/(cpm positive standard−cpm negative standard) × 1000). The cutoff value for identifying positive samples for autoantibodies against PDXP was 279. Lanes 1–14: sera from patients with tumours of different origins. 1: lymphoma, 2: mammary, 3: bladder, 4: ovary, 5: uterus, 6: testis, 7: skin (malignant melanoma), 8: prostate, 9: kidney, 10: colon, 11: bile duct, 12: rectum, 13: other, 14: lung. Lanes 15–18: sera from patients with other autoimmune diseases and controls. 15: Addison's disease or APS type II, 16: type 1 diabetes, 17: multiple sclerosis and 18: blood donors.

Altogether, 22 of 243 (9.1%) lung cancer patients and eight of 113 (7.1%) patients with various other types of cancer had autoantibodies against PDXP (Figure 4

), whereas two of 88 (2.3%) sera from healthy controls revealed reactivity (*P*=0.046 when comparing all cancer patients with healthy controls). In addition, one of 47 (2.1%) patients with type 1 diabetes (three of 72 (4.1%) patients with autoimmune Addison's disease or APS and two of 47 (4.5%) MS patients had autoantibodies against PDXP (Figure 4) (*P*=0.008 when comparing all four control groups with all cancer patients). Clinical data were only available from some of the cancer patients with PDXP antibodies, and no PND was discovered in these. Onconeuronal antibodies (anti-Hu) were detected in five of the anti-PDXP positive sera from the patients with lung cancer using immunofluorescence and immunoblot. None of the antibody-positive patients with Addison's disease, APS, diabetes or MS had known malignant disease. Among the patients with sera containing PDXP antibodies detected by RIA, five of 15 (33%) patients with lung cancer and three of eight (38%) patients with various types of cancer recognised PDXP by Western blot.

### Immunohistochemistry

**Figure 5:**
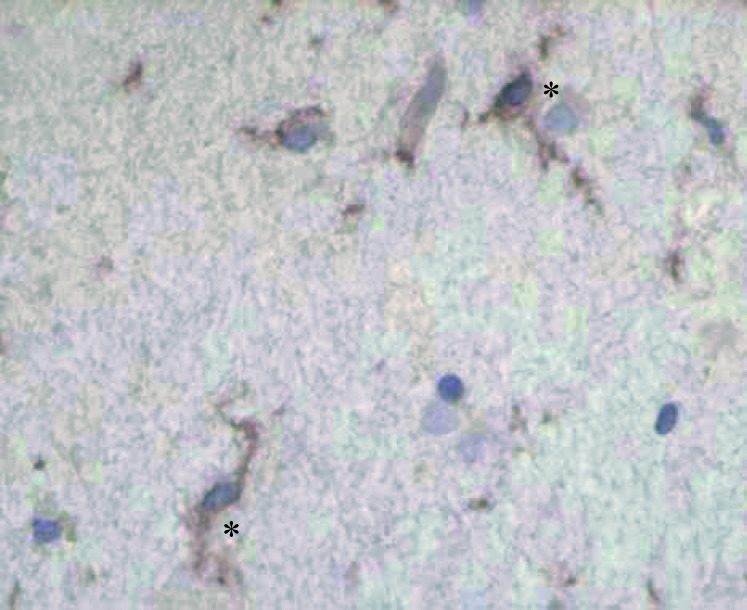
Anti-PDXP-stained cytoplasm of microglia of human brain sections in immunohistochemical experiments (400 ×). Examples of stained cells are marked with ^*^.

The rabbit antisera against PDXP stained human cerebrum and cerebellum but not liver, spleen, lung, heart, kidney and muscle (data not shown). Anti-PDXP stained the cytoplasm of microglia (Figure 5

) and astrocytes. Neurons also stained weakly.

## DISCUSSION

This study identified PDXP as a novel autoantigen associated with various types of cancer, including lung cancer. In total, 9% of patients with lung cancer and 7% of patients with other types of cancer revealed reactivity against PDXP *vs* 2% among healthy controls. In the immunoprecipitation experiments, some sera reacted with native proteins and failed to recognise proteins that had been frozen (data not shown). This may imply that the protein changed its conformation after freezing and thawing and that autoantibodies recognise conformational epitopes. This speculation is supported by the finding that only a subgroup of the patients positive in the RIA assay showed reactivity in Western blot. RIA seems to be a more sensitive method for detecting autoantibodies, similar to the findings with anti-Hu (Storstein *et al*, 2004) and antiinsulin (Sodoyez-Goffaux *et al*, 1988). Five of the lung cancer patients positive for anti-PDXP also had circulating Hu-antibodies but not clinically evident PND. Onconeuronal antibodies were not detected in any of the other anti-PDXP positive sera. Our findings therefore indicate that PDXP is associated with cancer and not with PND, but the latter association cannot be ruled out.

Autoantibodies against several different proteins are frequent in cancer patients, and most appear in a wide variety of types of cancer (Tan and Shi, 2003; Zhang *et al*, 2003), similar to our findings of anti-PDXP. Autoantibodies against the cell cycle checkpoint protein p53, for example, are found in 9–20% of human cancer patients with a specificity of 96% (Soussi, 2000). Hu and VGCC antibodies were not related to improved survival in patients with small-cell lung cancer (Monstad *et al*, 2004). The effects of circulating autoantibodies against PDXP are still unclear. Future studies on more well-defined patient materials should elucidate the sensitivity and specificity of the association between antibodies against PDXP and cancer. Our finding of an increased frequency among patients with Addison's disease, APS and MS compared with controls may imply other associations than cancer. It would also be interesting to determine whether anti-PDXP in patients affects the course and prognosis of malignant disease.

The recent study by Jang *et al* (2003) revealed that the expression of PDXP is ubiquitous, although with higher levels in the central nervous system, testis and liver. This is in agreement with our results. We found high expression in the central nervous system but also in a number of cancer cell lines, which could explain why PDXP becomes an autoantigen in patients with cancer. The PLP level in various tissues is regulated by several enzymes: PDXP, pyridoxal kinase, pyridoxamine-5′-phosphate oxidase and various binding proteins (Anderson *et al*, 1974; Li *et al*, 1974; Harris, 1990; Fonda, 1992). The brain contains an especially high concentration of PLP, and this is where the highest expression of PDXP is. More than 140 distinct enzymatic reactions depend on PLP according to the Enzyme Commission (http://www.chem.qmul.ac.uk/ium bm/enzyme) (Percudani and Peracchi, 2003), including many enzymes that participate in the biosynthesis of important neurotransmitters. Examples of the latter are glutamate decarboxylase participating in the synthesis of GABA and aromatic L-amino acid decarboxylase participating in the biosynthesis of catecholamines and serotonin. Interestingly, these enzymes have recently been found to be autoantigens in nervous system and endocrine diseases. Antibodies against glutamate decarboxylase (Lindefors, 1993) are found in type 1 diabetes (Baekkeskov *et al*, 1990), stiff-man syndrome (Solimena *et al*, 1990) and APS type I (Velloso *et al*, 1994). Autoantibodies against aromatic L-amino acid decarboxylase (Rorsman *et al*, 1995), histidine decarboxylase (Skoldberg *et al*, 2003) and cysteine sulfinate decarboxylase (Skoldberg *et al*, 2004) are all found in patients with APS type I. We have now shown that another enzyme related to PLP is an autoantigen, making PLP a common denominator for enzymes targeted by the immune system in endocrine and nervous system autoimmune diseases.

The reason why the immune system recognises PDXP and other PLP-associated autoantigens as non-self remains obscure. PLP, which is the coenzymatically active form of vitamin B_6_ (pyridoxine), is reported to suppress tumorigenesis, such as in colon cancer (Matsubara *et al*, 2003). The mechanisms behind the chemopreventive effect of vitamin B_6_ probably combine inhibitory effects on cell proliferation, oxidative stress and angiogenesis. One theory is that some tumours express elevated levels of PDXP to overcome the suppressive effect of vitamin B_6_. The increased expression might induce a break in immune tolerance against PDXP. The Multiple Tissue Expression Array revealed that some cancer cell lines express PDXP. PLP has also recently been suggested to have immunomodulatory properties because it binds very tightly to the D1 domain of CD4 on T cells, thereby interfering with proper interaction between CD4 and the major histocompatibility complex class II (Salhany and Schopfer, 1993). Lack of interaction leads to T-cell apoptosis and anergy to autoantigens (Namazi, 2003). One might speculate increased expression of PDXP degrade PLP, thereby facilitating tumour growth, but this could also lead to an enhanced immune response against the tumour.

In conclusion, PDXP is a novel autoantigen associated with cancer. PLP seems to be a common denominator for autoantigens involved in nervous system and endocrine autoimmune diseases. The clinical implications of anti-PDXP remain to be defined.
